# Disjunct distributions of freshwater snails testify to a central role of the Congo system in shaping biogeographical patterns in Africa

**DOI:** 10.1186/1471-2148-14-42

**Published:** 2014-03-06

**Authors:** Roland Schultheiß, Bert Van Bocxlaer, Frank Riedel, Thomas von Rintelen, Christian Albrecht

**Affiliations:** 1 Division of Genetics and Physiology, Department of Biology, University of Turku, Itäinen Pitkäkatu 4, 20014 Turku, Finland; 2 Department of Animal Ecology & Systematics, Justus Liebig University Giessen, Heinrich-Buff-Ring 26-32, Giessen 35392, Germany; 3 Departments of Paleobiology and Invertebrate Zoology, National Museum of Natural History, Smithsonian Institution, Washington, DC 20013, USA; 4 Research Unit Palaeontology, Department of Geology and Soil Science, Ghent University, Krijgslaan 281 S8, Ghent 9000, Belgium; 5 Institute of Geological Sciences, Freie Universität Berlin, Malteserstr 74-100, Berlin 12249, Germany; 6 Key Laboratory of Plateau Lake Ecology and Global Change, Yunnan Normal University, No. 1 Yuhua District, Chenggong, Kunming, China; 7 Museum für Naturkunde, Leibniz-Institut für Evolutions- und Biodiversitätsforschung an der Humboldt-Universität zu Berlin, Invalidenstrasse 43, Berlin 10115, Germany

**Keywords:** Freshwater biogeography, East African Rift System, Congo, Lake Malawi, Lake Tanganyika, Zambezi

## Abstract

**Background:**

The formation of the East African Rift System has decisively influenced the distribution and evolution of tropical Africa’s biota by altering climate conditions, by creating basins for large long-lived lakes, and by affecting the catchment and drainage directions of river systems. However, it remains unclear how rifting affected the biogeographical patterns of freshwater biota through time on a continental scale, which is further complicated by the scarcity of molecular data from the largest African river system, the Congo.

**Results:**

We study these biogeographical patterns using a fossil-calibrated multi-locus phylogeny of the gastropod family Viviparidae. This group allows reconstructing drainage patterns exceptionally well because it disperses very poorly in the absence of existing freshwater connections. Our phylogeny covers localities from major drainage basins of tropical Africa and reveals highly disjunct sister-group relationships between (a) the endemic viviparids of Lake Malawi and populations from the Middle Congo as well as between (b) the Victoria region and the Okavango/Upper Zambezi area.

**Conclusions:**

The current study testifies to repeated disruptions of the distribution of the Viviparidae during the formation of the East African Rift System, and to a central role of the Congo River system for the distribution of the continent’s freshwater fauna during the late Cenozoic. By integrating our results with previous findings on palaeohydrographical connections, we provide a spatially and temporarily explicit model of historical freshwater biogeography in tropical Africa. Finally, we review similarities and differences in patterns of vertebrate and invertebrate dispersal. Amongst others we argue that the closest relatives of present day viviparids in Lake Malawi are living in the Middle Congo River, thus shedding new light on the origin of the endemic fauna of this rift lake.

## Background

The formation of the East African Rift System (EARS) played a decisive role in the evolution of Africa’s tropical fauna and flora. The climatic, geological, and hydrological consequences of the rifting processes are critical to understand the emergence of the endemic species of the savannahs and the Great Lakes as well as the origin of humankind. Rifting created, disrupted, redirected, and connected major freshwater systems [1].

**Figure 1:**
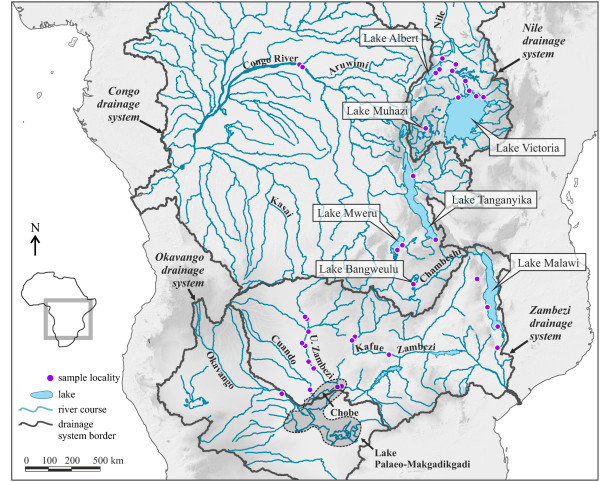
**Map of tropical Africa with major drainage systems.** The map shows the major drainage systems, framed with bold grey lines, and the sampling points of the study. Lakes and rivers referred to in the text are labelled accordingly; the Upper Zambezi is abbreviated as ‘U. Zambezi’. The dashed outline indicates the maximum expansion of Lake Palaeo-Makgadikgadi, modified after Riedel et al. [4]. Locality details for each sampling point are given in Table 1.

The three largest drainage systems in present-day Central Africa are the Congo-, the Nile-, and the Zambezi system (Figure 1). The Congo River covers an area of ~3.7 million square kilometres and has a length of ~4100 kilometres [2]. It drains part of the EARS (including Lake Tanganyika) and borders to the Nile system in the northeast. The Nile system receives water from Lake Albert and Lake Victoria and drains a region of ~3.2 million square kilometres for almost 7000 kilometres northwards to the Mediterranean Sea. To the south, the Congo system borders to the Zambezi drainage system, which runs ~2600 kilometres, covers an area of 1.3 million square kilometres [2,3], and includes the southernmost of the Great Lakes, Malawi. The central location of the EARS to these river systems bears witness to the rift’s importance in shaping drainage pattern of tropical Africa, but their history since the Mid-Miocene is only partly resolved and arguably complex [2,3].

Most suggestions of palaeohydrological connections in tropical Africa are based on circumstantial evidence in combination with general models of rifting and uplifting [2] rather than on geomorphological evidence like in other parts of the continent where palaeochannels can be traced over considerable areas and are reasonably well dated, e.g. [5-7]. The consequential lack of a coherent synthesis or a model of hydrographical connections since the Mid-Miocene severely hampers our understanding of historical biogeography in tropical Africa. This is further complicated by a scarcity of samples from the Congo River system, e.g. [8-11], but see Goodier et al. [12] and Day et al. [13]. The Congo is the largest freshwater system in the centre of the continent and palaeohydrological research indicates that the river and its tributaries have a highly vicissitudinous drainage history. Temporal connections existed to the present day Lake Victoria area [14,15], Lake Turkana [16,17], the Upper Zambezi area [18,19] and the Indian Ocean [19], although the latter connection is controversial [1]. Hence, the analysis of Congolese samples is a prerequisite for our understanding of the evolution and distribution of the freshwater fauna of tropical Africa—including the origins of the endemic radiations in the rift-lakes Malawi and Tanganyika.

Here we study the historical biogeography of the freshwater gastropod family Viviparidae, a taxon widely distributed in the lotic and lentic environments of tropical Africa [20]. This family is of particular interest to studies of historical freshwater biogeography because its dispersal is mostly limited to habitats with existing freshwater connections [21], rendering it an ideal tracer of past and present hydrographic connections. The ovoviviparous and dioecious reproduction mode of viviparids minimizes opportunities for successful vector-mediated migration and a subsequent establishment of a viable population [22,23]. Accordingly, viviparid fossils are conspicuously absent from African water bodies that were never hydrographically connected to a source, e.g. crater- and oasis lakes [21].

We obtained samples from the major drainage systems in the region, i.e. that of the Nile, the Okavango, the Zambezi as well as the Upper and Middle Congo. Using Bayesian multi-locus phylogenetic analyses and a fossil-calibrated, relaxed molecular clock we studied the diversification and distribution of viviparids in Africa. In light of the impact of the evolution of the EARS on African hydrographic systems and the dependence of our model organism on these systems to disperse, we expect a deep phylogenetic separation between viviparid species east and west of the western branch of the rift system. We furthermore hypothesize this separation to temporarily coincide with the disruption of freshwater migration routes across the evolving rift.

## Results

### Phylogenetic analyses

**Figure 2:**
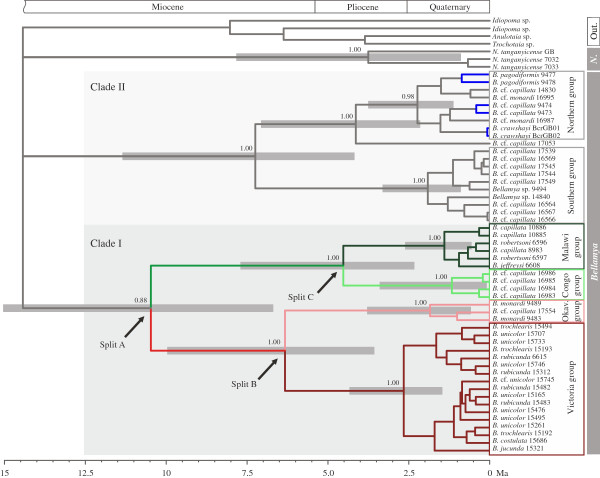
**Multi-locus molecular phylogeny of the African Viviparidae.** The phylogeny comprises the genera *Bellamya* and *Neothauma* (abbreviated as ‘*N*.’). The phylogeny was dated using a fossil-calibrated uncorrelated lognormal clock model. Clade I consists of four colour-coded, geographically distinct subgroups from the Middle Congo, Lake Malawi, the Lake Victoria area and the Okavango area. Colours correspond to those in Figure 3. Clade II consists of a Northern group with specimens from lakes Mweru and Bangweulu (highlighted in blue) and from the Northern Kafue as well as a Southern group with specimens from the Upper Zambezi, the Chobe, and the Southern Kafue. Confidence intervals (95%) for age estimates of selected nodes are indicated by grey bars; Bayesian posterior probabilities are given for selected nodes; ‘Out.’ indicates the outgroup. Locality details are provided in Table 1.

The Bayesian analyses of the African Viviparidae yielded one well-supported *Neothauma* group from Lake Tanganyika (Figure 2; Bayesian posterior probabilities [BPP] 1.0) and two *Bellamya* clades comprising specimens from the sampled major drainage systems of tropical Africa (Clades I and II in Figure 2; BPP 0.88 and 1.0). Separate analyses of the nuclear and the mitochondrial dataset revealed no phylogenetic incongruences (Additional file 1). Clade I contains four well-supported, reciprocally monophyletic groups of specimens from (i) lakes Albert and Victoria and the Nile River (‘Victoria group’; dark red in Figure 2; BPP 1.0); (ii) the Middle Congo River (‘Congo group’; light green in Figure 2; BPP 1.0); (iii) the rivers Chobe/Cuando and Okavango (‘Okavango group’; light red in Figure 2; BPP 1.0) and (iv) the Malawi Basin (‘Malawi group’; dark green in Figure 2; BPP 1.0). Within Clade I the Congo group is sister to the Malawi group whereas the Victoria group is sister to the Okavango group (both sister-group relationships are supported with BPP 1.0). Clade II consists of a Northern group with specimens from lakes Mweru and Bangweulu (highlighted in blue in Figure 2) and from the Northern Kafue (BPP 0.98) as well as a Southern group with specimens from the Upper Zambezi, the Chobe, and the Southern Kafue (BPP 1.0). Due to lack of support in the two deepest nodes of the phylogeny (BPP < 0.7) we can make no conclusive statement as to the monophyly of either the African *Bellamya* or the African Viviparidae. These nodes were collapsed into a polytomy in Figure 2. The number of parsimony informative sites and effective sampling sizes are provided in Additional file 1 for each fragment separately.

The phylogenetic structure revealed by Figure 2 also indicates that morphological identifications and genealogy do not match each other perfectly. A main concern is the systematic position of *Bellamya capillata*, which has been traditionally reported from many localities in Africa south of the equator [20]. The type locality for this taxon is Lake Malawi, and all other localities from which the species was identified, are in fact inhabited by populations that are deeply divergent from *B. capillata* in Lake Malawi. Similar issues exist for other *Bellamya* species (see Discussion). We thus tentatively name specimens with an ambiguous status in current systematics using ‘confer’ (cf.) to emphasize that taxonomic revisions are required.

### Molecular clock estimates

We focus on estimates for the ages of the following three nodes in Clade I (Figure 2): Split A—most recent common ancestor (mrca) of the entire clade: 10.47 million years (My) (95% confidence interval [CI]: 6.69-15.04 My); Split B—the split between the Victoria- and the Okavango group: 6.32 My (CI: 3.58-9.96 My); Split C—the split between the Congo- and the Malawi group: 4.52 My (CI: 2.32-7.70 My). The age of the mrca of the Southern group in Clade II is 1.916 My (CI: 0.88-3.31 My). Note that the here estimated mean substitution rate of the COI fragment is 0.93% per My (CI: 0.68-1.18) and therewith very similar to the trait-specific COI Protostomia clock rate for the HKY model suggested by Wilke et al. [24] for dioeceous tropical and subtropical taxa with a generation time of 1 year and a body size of approximately 2–50 mm (mean rate: 1.24% per My; CI: 1.02-1.46). Substitution rates are provided in Additional file 1 for each fragment.

## Discussion

**Figure 3:**
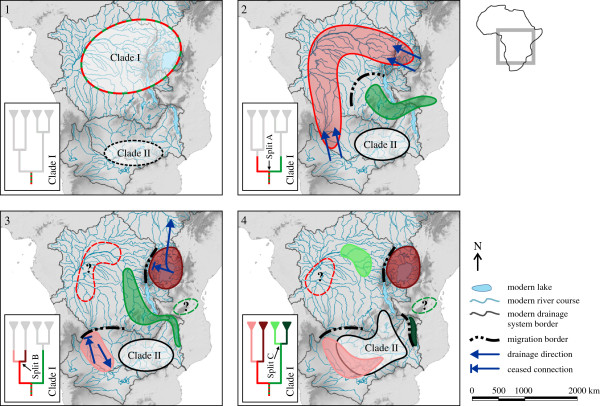
**Biogeographical model for *Bellamya*.** The model comprises four progressive stages covering roughly **(1)** Middle Miocene, **(2)** Late Miocene, **(3)** Pliocene and **(4)** Pliocene/Early Pleistocene timeframes. The map shows the current borders and courses of the drainage systems and rivers as well as the current sizes of the lakes. Inset phylogenies track the diversification of Clade I: colours correspond to geographic ranges and the phylogenetic groups in Figure 2. The borders of the suggested ranges are approximations; detailed descriptions of the individual stages are given in the text. Question marks within the dashed areas in stages 3 and 4 indicate potential remnant populations of the clusters from stage 2.

The phylogenetic reconstruction reveals two, geographically highly disjunct sister-group relationships in Clade I (Figures 2 and 3-4): the Congo-Malawi-cluster and the Victoria-Okavango-cluster. The groups within each cluster are separated by at least one water divide. This conspicuous spatial pattern stands in sharp contrast to the initially stated expectation that species east of the western branch of the EARS are closer related to each other than to species west of it, and vice versa. Vector-mediated dispersal across water divides is an unlikely explanation for the observed biogeographical pattern for reasons mentioned earlier. Moreover, given the wide spatial overlap of the potential dispersal corridors of the two clusters, we would expect to observe population exchange between taxa from e.g. the Middle Congo and the Nile, or between the Malawi- and the Zambezi/Okavango group, because the involved vectors would have been able to operate in the entire area. Yet the reciprocal monophyletic clades within our phylogeny argue clearly against such an exchange.

In order to explain our findings we integrated our phylogenetic information with literature data on palaeohydrographical connections to provide a synthesized, spatially and temporarily explicit model of historical freshwater biogeography in tropical Africa. Subsequently, we review recently published studies on African fish biogeography and evaluate similarities and dissimilarities of vertebrate and invertebrate dispersal patterns.

### Historical biogeography of *Bellamya* in tropical Africa

Changes in the structural geology of East Africa associated with uplifting and rifting [25] as well as pronounced moisture cycles over the past 5 My—mainly governed by orbitally-induced insolation at low altitudes [26,27]—have created a dynamic setting for palaeohydrographical connections and, hence, organismal dispersal. Against this background, we propose the following biogeographical model, which explains the two, spatially disjunct sister-taxon relationships as a consequence of successive changes in hydrographical connections between the Congo River system and its neighbouring water systems (stages 1–4 in Figure 3; refer to Figure 1 for names of water bodies):

(1) The earliest African viviparid fossils were found in tropical regions [15] (Additional file 2). We thus assume that this region is the geographical origin of an ancestral population of Clade I in the Middle/Late Miocene (~12 Ma; Figure 3-1).

(2) Subsequently this ancestral lineage split into the Congo-Malawi- and the Victoria-Okavango-cluster (Split A in Figures 2 and 3-2; mean age ~10.5 My). In the Late Miocene/Early Pliocene the waters of the present day Lake Victoria region drained via a precursor of the Aruwimi River into the Congo [14,15]. A contemporaneous connection from the Congo system to the headwaters of the Upper Zambezi was suggested via the Kasai River [19,28,29], which served as a dispersal route for various fish taxa [11,12,30,31]. This persistent freshwater connection likely also enabled viviparid migration between the present day Lake Victoria region and the Upper Zambezi/Okavango watershed (Figure 3-2). We postulate that contemporaneously the Congo-Malawi-cluster inhabited the Upper Congo catchment including the Chambeshi system—which drained eastward into the Rufiji system until the Pliocene [19]—, the emerging Lake Malawi, and perhaps Palaeolake Rukwa [32] (Figure 3-2). At that time no hydrographical connection existed between the Upper and the Middle Zambezi [28,33,34] and there was no faunal exchange between the Victoria-Okavango-cluster and the Congo-Malawi-cluster.

(3) In the Late Miocene/Early Pliocene the Okavango group started to separate from the Victoria group (Split B in Figures 2 and 3-3; mean age ~6.3 My). During a wet phase in East Africa around 3.0-2.8 Ma faunal elements of Congolese origin (e.g. molluscs and sponges) colonised the Turkana Basin for the last time and all subsequent invasions into the basin were of Nilotic origin [16,17]. This data indicates that the connection of the Victoria region to the Central Congo ceased, at latest with the closure of the Beni Gap in the Early Pleistocene [15] but perhaps earlier [1]; a similar timeframe is assumed for the disruption of the Okavango/Upper Zambezi-Congo connection [3,28,35]. We argue that the final separation of the two groups was ultimately governed by these events (Split B in Figure 2) but we acknowledge that the confidence interval for Split B also allows for an earlier separation. Around this time, the ancestors of the current Congo-Malawi groups inhabited an area encompassing the Upper Congo system, the Chambeshi system, and young Lake Malawi, most likely using the northwest corner of the lake’s basin as gateway [3,19] (Figure 3-3).

(4) Subsequently the connection between Lake Malawi and the Chambeshi/Upper Congo system ceased, which separated the Congolese from the Malawian *Bellamya* (Split C in Figures 2 and 3-4; mean age ~4.5 My). This faunal disruption may have been a direct consequence of rifting in the Mweru-Mweru Wantipa fault zone and in the northern part of the Malawi Rift [36-38]. The extant viviparid fauna of the lakes Mweru and Bangweulu is however no remnant of this former Congo-Malawi-cluster (Figure 2). In fact, age and phylogenetic position of the Mweru/Bangweulu viviparids suggest a re-colonization of the two lakes from the Zambezi via the Kafue River [39], perhaps after a climatically-induced Pleistocene ecosystem crisis comparable to those in nearby Lake Malawi in the east [40-42] and Lake Palaeo-Makgadikgadi to the west [43]. Moreover, the polyphyletic pattern of the Mweru/Bangweulu viviparids indicates that the two lakes may have been colonized multiple times from the Zambezi River system (Figures 2 and 3-4). In the Late Pliocene/Early Pleistocene the Upper Zambezi was captured by the Middle Zambezi [28,34], which enabled secondary contact between the Okavango group and Clade II (Figures 2 and 3-4). The observable geographical overlap between the two groups coincides with the area of Lake Palaeo-Makgadikgadi, which existed in consecutive phases over the Middle and Late Pleistocene [43-45] (Figures 1 and 3-4). This lake may have stirred populations of initially different geographic origin (e.g. the Okavango group and Clade II) and, hence, superimposed previous spatial patterns resulting in the current overlapping distributions of *Bellamya* groups.

### Lake Malawi

The phylogeny presented here reveals an unanticipated sister relationship of Lake Malawi’s extant *Bellamya* fauna to that of the Middle Congo. In light of the well-supported reciprocal monophyly of both groups we argue that the separation of the Congo-Malawi-cluster (Split C in Figures 2 and 3-4) was a consequence of the disruption of hydrological connections and therewith migration routes (e.g. due to rifting processes) rather than of dispersal from the Congo River system into Lake Malawi or vice versa. Dispersal would most likely have resulted in a pattern where the sink population is nested within the paraphyletic source population instead of the observed reciprocal and strict regional monophyly. The *Bellamya* of the lakes Mweru and Bangweulu, which re-colonized these water bodies from the Zambezi system, illustrate such a case of dispersal (Figure 2). We hence also conclude that the general dominance of regionally confined monophyletic clusters in our dataset points toward a prevalence of vicariance over dispersal processes in the historical biogeography of the taxon—in contrast to, e.g., some fish taxa (see below).

Viviparid fossils with an age of up to four million years are reported from the Chiwondo Beds in the Northwest of the Malawi Basin [46]. Because the mean age of the split between the Malawi and the Congo group is of a similar age, our results do neither reject the possibility of an uninterrupted evolutionary *Bellamya* lineage in Lake Malawi (the founder-flush model in Schultheiß et al. [47,48]) nor the possibility that the lake was re-colonized after its Pliocene fauna went extinct, e.g. during severe lake level low stands [41]. In any case, the onset of molecular diversification of the modern endemic Malawi group started considerably later (1.39 Ma; CI: 0.56-2.61 My) than the deposition of the Plio-Pleistocene Chiwondo Bed fossils. Thus it has paralleled the diversification of the endemic *Lanistes* taxa of Lake Malawi [48].

### Lake Tanganyika

Although *Neothauma* is currently endemic to Lake Tanganyika, this has not been the case from the Late Miocene to the Early Pleistocene when the genus radiated in Palaeolake Obweruka [15]. This palaeolake occupied the Lake Albert-Edward region and was similar in size and depth to Lake Tanganyika prior to the uplift of the Rwenzori Mountains ~2.3 Ma [14,49]. The molecular phylogeny indicates that the *Neothauma* lineage from Lake Tanganyika evolved fairly isolated from the African *Bellamya* (Figure 2). A similar spatial, temporal, and genetic distinctness is reported for some fish taxa of Lake Tanganyika [31,50,51]. It appears reasonable to assume that the lake served as a refuge and harbours the sole survivors of the formerly more widely distributed and morphologically diverse genus *Neothauma*. Moreover, the isolated evolutionary history of Lake Tanganyika’s viviparid fauna implies that for the timeframe and migration events discussed in this paper the lake served neither as a gateway nor as a dispersal route.

### Comparative biogeography of afrotropical freshwater taxa

This paper is to our knowledge the first invertebrate study investigating patterns of historical freshwater biogeography in tropical Africa using molecular data. Against the background of recent studies on African fish biogeography we here review patterns of vertebrate and invertebrate biogeography with a strong focus on dispersal abilities, hybridization, and migration routes.

A striking difference between viviparid and fish patterns lies in the phylogenetic relationships of taxa from the modern Lake Victoria region: fish specimens from the Lake Victoria region cluster generally with other East African species, e.g. [31,50,52,53]. In contrast, the sister-taxon relationship between viviparids from the Lake Victoria region and those from the Okavango/Upper Zambezi is to date unique among the afrotropical freshwater fauna. This pattern is remarkable because connecting these two drainage systems requires crossing two divides: (A) the Congo/Upper Zambezi water divide via a precursor of the Kasai River and (B) crossing the emerging western branch of the EARS. To provide a hypothesis as to the cause of the difference of biogeographical patterns between fish and viviparid taxa, we suggest that the rifting process was the vicariance event that caused the main phylogenetic separation of fish taxa into species east and west of the western branch of the EARS. At this stage, however, the afrotropical Viviparidae were already separated into the Congo-Malawi cluster and the Victoria-Okavango cluster (in the aftermath of Split A; Figures 2 and 3). We argue that this initial Split A was invoked by a migration barrier—perhaps in the Congo Basin—that inhibited viviparid dispersal yet allowed fish dispersal. Intrinsic biological properties may create differential abilities to overcome ecological, behavioural, and physical barriers [30], e.g. differences in mobility may cause differential migration through corridors which only exit during annual flooding. For example, two major flooding events since 2010 promoted the migration of fish from the Okavango Delta into the formerly dry Boteti river bed. However, *Bellamya*—which lives in permanent overspills of the Okavango Delta such as the Thamalakane River—did not migrate into the Boteti River (FR pers. observation, 2010, 2011, and 2013).

Eventually, the rifting process separated the two viviparid clusters into the four modern groups (i.e. the Congo-, Malawi, Okavango-, and Victoria group; Splits B and C) subsequently to Split A. Each of these groups is (much like in fish) confined to one side of the western branch of the EARS.

This pattern and comparative biogeography sheds new light on freshwater migration corridors over the emerging rift. African tigerfish, for example, were suggested to have migrated during the Late Miocene along an east–west corridor from the Tanzanian Rufiji-Ruaha system via the Luangwa to the Upper Zambezi and the Okavango [12], crossing the young rift at the narrow Rukwa-Rungwe region prior to the uplift of the Ufipa plateau [54]. A similar corridor over the rift appears to have been feasible for mastacembelid eels [51] and, in fact, also for viviparids: The Congo-Malawi-cluster would likewise have occupied the Rukwa-Rungwe region (green area in Figure 3-2). It would hence be highly improbable that members of the Victoria-Okavango cluster migrated contemporaneously from the Victoria region through this corridor across the Rukwa-Rungwe area into the Upper Zambezi/Okavango. This would imply that members of this cluster migrated along the same hydrographical system as the members of the Congo-Malawi-cluster, yet without disrupting the here revealed biogeographical pattern. Consequently, we suggest the existence of a second east–west migration corridor (red area in Figure 3-2) connecting the Victoria region with the Okavango/Upper Zambezi area.

With the exception of some branches in Clade II, our phylogeny generally features strict regional monophyly—a pattern often absent in biogeographical studies of fish in tropical Africa, e.g. [11,12,55]. This absence is generally attributed to hybridization and active, long distance dispersal, e.g. [8,11,12,31,52], which hence appear to have played only a minor role in the historical biogeography of viviparids. Therewith, superimposition of underlying distributional patterns by recent active dispersal or hybridization seems negligible for this gastropod group, which in turn facilitates the reconstruction of original migration corridors.

The decisive phylogenetic pattern notwithstanding, further sampling is required to substantiate the proposed model. In particular, more sampling from the central Congo area is desirable despite logistic challenges, e.g., to search for putative remnants of the Victoria-Okavango-cluster (red dashed area in Figure 3-3). Furthermore, specimens are required from the Rufiji-Ruaha system to test our hypotheses associated with the Malawi-Congo-cluster (green dashed area in Figure 3-3). Eventually, more comprehensive sampling of the Lower Zambezi may shed light on the origins and evolution of Clade II. It is noteworthy that such an extension of the dataset will also enable a systematic revision of the genus *Bellamya*, as our work reported considerable incongruences between traditional taxonomy and the results of molecular phylogenetic analyses, e.g. [47,56].

## Conclusions

We have presented a first integrated model of historic freshwater invertebrate biogeography in tropical Africa. By comparing our results to fish data we demonstrate the importance of broad geographical and taxonomic sampling encompassing taxa with different dispersal abilities and life history traits. Our analysis shows that the biographical history of viviparids in tropical Africa is driven by consecutive vicariance events rather than by dispersal and is closely linked to the formation of the EARS. Moreover, we demonstrate that the Congo drainage system plays a central role in the distribution of the continent’s freshwater fauna during the late Cenozoic. Our data also sheds new light on the geographic origin of the endemic fauna of Lake Malawi and reveals its close phylogenetic relationship to Congolese populations. We hope that future work, with a further inclusion of invertebrate taxa, will help to refine our understanding of biogeographical patterns of tropical Africa’s freshwater fauna.

## Methods

### Sampling and species identification

Specimens of Viviparidae were collected between 2006 and 2012 in tropical Africa (Figure 1; Table 1). There are two extant genera of the family on the continent: the widely distributed *Bellamya* and the monospecific *Neothauma*, which is endemic to Lake Tanganyika [20]. Due to the ambiguous phylogenetic relationship between the two genera [47,56], we furthermore added the following closely related Asian viviparid genera to our dataset: *Anulotaia*, *Idiopoma*, and *Trochotaia*. Material was identified before molecular analysis based on shell morphology following the currently accepted taxonomy of African Viviparidae, e.g. [20].

**Table 1:** Locality details and NCBI GenBank accession numbers of the specimens analysed in this study

**DS**	**Water body**	**Country**	**Longitude**	**Latitude**	**Species**	**Sp. ID**	**Voucher ID**	**COI**	**mtLSU**	**ncLSU**	**H3**
Congo	Lake Tanganyika	Tanzania	29.69946	−4.78543	*N. tanganyicense*	7032*	ZMB113503	HQ012716	HQ012683	HQ012706	
Congo	Lake Tanganyika	Tanzania	29.69946	−4.78543	*N. tanganyicense*	7033*	ZMB113504	HQ012717		HQ012707	
Congo	Lake Tanganyika	Zambia	n/a	n/a	*N. tanganyicense*	GB^#^		FJ405843	FJ405709	FJ405598	FJ405739
Congo	Congo River	Congo	22.66692	2.09519	*B.* cf. *capillata*	16983	ZMB 113784			JX489348	JX489282
Congo	Congo River	Congo	22.66347	2.09981	*B.* cf. *capillata*	16984	ZMB 113785			JX489349	JX489283
Congo	Congo River	Congo	22.66347	2.09981	*B.* cf. *capillata*	16985	ZMB 113786	JX489246	JX489315	JX489350	JX489284
Congo	Congo River	Congo	22.66347	2.09981	*B.* cf. *capillata*	16986	ZMB 113787	JX489247		JX489351	JX489285
Congo	Lake Bangweulu	Zambia	29.70750	−11.45268	*B.* cf. *capillata*	14830	ZMB 113788	JX489224	JX489295	JX489328	
Congo	Lake Mweru	Zambia	29.04460	−9.04567	*B.* cf. *capillata*	9473*	ZMB 113515	HQ012725	HQ012692	JX489326	JX489259
Congo	Lake Mweru	Zambia	29.04460	−9.04567	*B.* cf. *capillata*	9474*	ZMB 113512	HQ012722	HQ012690		JX489260
Congo	Lake Mweru	Zambia	28.73133	−9.34801	*B. pagodiformis*	9477*	ZMB 113510	HQ012720	HQ012688		JX489261
Congo	Lake Mweru	Zambia	28.73133	−9.34801	*B. pagodiformis*	9478*	ZMB 113511	HQ012721	HQ012689	JX489327	
Congo	Lake Mweru	Zambia	n/a	n/a	*B. crawshayi*	GB01^#^		FJ405844	FJ405695	FJ405596	FJ405746
Congo	Lake Mweru	Zambia	n/a	n/a	*B. crawshayi*	GB02^#^		FJ405867	FJ405710	FJ405591	
Nile	Nile River	Uganda	33.15524	0.48544	*B. unicolor*	15165	ZMB 113789	JX489226	JX489297	JX489330	
Nile	Nile River	Uganda	31.50487	2.45933	*B. unicolor*	15476	ZMB 113790	JX489232	JX489303	JX489336	JX489270
Nile	Nile River	Uganda	32.93648	1.09002	*B. unicolor*	15707	ZMB 113791	JX489238	JX489307	JX489341	JX489275
Nile	Nile River	Uganda	32.09473	1.69255	*B. unicolor*	15733	ZMB 113792	JX489239	JX489309	JX489342	JX489276
Nile	Nile River	Uganda	32.33107	2.12373	*B. unicolor*	15746	ZMB 113793	JX489241		JX489344	
Nile	Lake Albert	Uganda	30.91564	1.44854	*B. rubicunda*	6615*	ZMB 113501	HQ012711	HQ012681	HQ012705	
Nile	Lake Albert	Uganda	31.10057	1.58649	*B. rubicunda*	15312	ZMB 113794	JX489230	JX489301	JX489334	JX489268
Nile	Lake Albert	Uganda	31.32021	1.81842	*B. rubicunda*	15482	ZMB 113795	JX489233	JX489304	JX489337	JX489271
Nile	Lake Albert	Uganda	31.32021	1.81842	*B. rubicunda*	15483	ZMB 113796	JX489234	JX489305	JX489338	JX489272
Nile	Lake Muhazi	Rwanda	30.47826	1.84843	*B.* cf. *unicolor*	15745	ZMB 113797	JX489240	JX489310	JX489343	JX489277
Nile	Lake Victoria	Uganda	32.49402	0.03892	*B. jucunda*	15321	ZMB 113798	JX489231	JX489302	JX489335	JX489269
Nile	Lake Victoria	Uganda	33.58264	0.16176	*B. trochlearis*	15192	ZMB 113799	JX489227	JX489298	JX489331	JX489265
Nile	Lake Victoria	Uganda	33.58264	0.16176	*B. trochlearis*	15193	ZMB 113800	JX489228	JX489299	JX489332	JX489266
Nile	Lake Victoria	Uganda	33.60258	0.14067	*B. unicolor*	15261	ZMB 113801	JX489229	JX489300	JX489333	JX489267
Nile	Lake Victoria	Uganda	32.49402	0.03892	*B. trochlearis*	15494	ZMB 113802	JX489235		JX489339	JX489273
Nile	Lake Victoria	Uganda	32.49402	0.03892	*B. unicolor*	15495	ZMB 113803	JX489236	JX489306		JX489274
Nile	Lake Victoria	Kenya	34.02031	0.1091	*B. costulata*	15686	ZMB 113804	JX489237	JX489307	JX489340	
Okavango	Okavango River	Namibia	21.58754	−18.12071	*B. monardi*	9489*	ZMB 113507	HQ012800	HQ012686	HQ012709	JX489263
Zambezi	Zambezi River	Zambia	23.08752	−13.52859	*B.* cf. *capillata*	16564	ZMB 113805	JX489242	JX489311		JX489278
Zambezi	Zambezi River	Zambia	23.23285	−14.38287	*B.* cf. *capillata*	16566	ZMB 113806	JX489243	JX489312	JX489345	JX489279
Zambezi	Zambezi River	Zambia	23.23285	−14.38287	*B.* cf. *capillata*	16567	ZMB 113807	JX489244	JX489313	JX489346	JX489280
Zambezi	Wanginga River	Zambia	22.84252	−15.08967	*B.* cf. *capillata*	16569	ZMB 113808	JX489245	JX489314	JX489347	JX489281
Zambezi	Kafue River	Zambia	28.20085	−15.81976	*Bellamya* sp.	14840	ZMB 113809	JX489225	JX489296	JX489329	
Zambezi	Kafue River	Zambia	26.04066	−14.81798	*B.* cf. *monardi*	16987	ZMB 113810	JX489248	JX489316	JX489352	JX489286
Zambezi	Kafue River	Zambia	26.10316	−14.70344	*B.* cf. *monardi*	16995	ZMB 113811	JX489249	JX489317	JX489353	JX489287
Zambezi	Zambezi River	Zambia	23.09447	−13.55640	*B.* cf. *capillata*	17053	ZMB 113812	JX489250	JX489318	JX489354	JX489288
Zambezi	Zambezi tributary	Zambia	22.95794	−15.19352	*B.* cf. *capillata*	17539	ZMB 113813	JX489251	JX489319	JX489355	JX489289
Zambezi	Zambezi River	Zambia	23.24116	−16.24204	*B.* cf. *capillata*	17544	ZMB 113814	JX489252	JX489320	JX489356	JX489290
Zambezi	Zambezi River	Zambia	23.24116	−16.24204	*B.* cf. *capillata*	17545	ZMB 113815	JX489253	JX489321	JX489357	JX489291
Zambezi	Zambezi, River	Zambia	23.56652	−16.65075	*B.* cf. *capillata*	17549	ZMB 113816	JX489254	JX489322	JX489358	JX489292
Zambezi	Zambezi River,	Zambia	25.186540	−17.75630	*B.* cf. *capillata*	17554	ZMB 113817	JX489255		JX489359	JX489293
Zambezi	Cuando River	Namibia	23.33815	−17.98192	*Bellamya* sp.	9494*	ZMB 113518	HQ012728	HQ012695		JX489264
Zambezi	Lake Kazuni	Malawi	33.64470	−11.14642	*B. capillata*	10885*	ZMB 113524	HQ012741			
Zambezi	Lake Kazuni	Malawi	33.64470	−11.14642	*B. capillata*	10886*	ZMB 113525	HQ012742			
Zambezi	Lake Malawi	Malawi	34.93017	−14.08923	*B. robertsoni*	6597*	ZMB 113584	HQ012797	HQ012704	JX489324	JX489257
Zambezi	Lake Malawi	Malawi	34.93017	−14.08923	*B. robertsoni*	6596*	ZMB 113574	HQ012752	HQ012698	JX489323	JX489256
Zambezi	Lake Malawi	Malawi	34.29794	−12.88355	*B. jeffreysi*	6608*	ZMB 113567	HQ012735	HQ012700	JX489325	JX489258
Zambezi	Shire River	Malawi	34.90498	−15.38884	*B. capillata*	8983*	ZMB113545	HQ012734	HQ012699	HQ012711	
Zambezi	Chobe River	Botswana	25.12985	−17.81595	*B. monardi*	9483*	ZMB 113508	HQ012718	HQ012687		JX489262

Taxonomic remarks: *Bellamya capillata* is reported to be widely distributed in the Zambezi system [20] but previous studies showed that the species is endemic to Lake Malawi [47,56]. We thus named morphologically similar specimens from elsewhere *B.* cf. *capillata* as a taxonomic revision is beyond the scope of this paper. Abbreviations: DS—drainage system; Sp. ID—specimen identity number; n/a—data not available; *specimen from Schultheiß et al. [47]; ^#^specimen from Sengupta et al. [56]; ZMB—Mollusca collection of the Berlin Museum of Natural History.

### DNA extraction and amplification

We used a CTAB protocol for DNA extraction [57] and amplified four DNA fragments: the cytochrome oxidase subunit I (COI) with universal [58] and specific primers [47], the mitochondrial large subunit of ribosomal RNA (LSU rRNA) [59], a fragment of the nuclear LSU rRNA [60], and the nuclear histone 3 [61]. PCR conditions were as given in [62] and sequencing was performed on an ABI3730XL sequencer (LGC Genomics Germany, Berlin). Sequences of all fragments were aligned unambiguously with ClustalW 1.4 [63] in BioEdit 7.1 [64] and checked by eye.

### Phylogenetic analyses and molecular clock estimations

We ran five independent Bayesian phylogenetic reconstructions (partitioned into the four DNA fragments) in BEAST 1.75 [65] to ensure convergence on the same posterior probability distribution. An unlinked HKY + Γ substitution model was utilized because more complex models showed indications of overfitting in preliminary runs; as tree prior we used a Yule model. In order to estimate divergence times we employed a fossil-based calibration using the earliest bellamyinid viviparid from Africa and the oldest *Neothauma* fossil (for an in-depth discussion see Additional file 2). The oldest *Bellamya* occurs in the Iriri Member of the Napak Formation at Napak and is ~19 My old [66]. To implement this calibration point, we used a uniform prior over the root of the phylogeny spanning 23–13 My to account for dating uncertainty and the various published hypotheses as to when the Bellamyinae invaded Africa from Asia, e.g. [66-68]. The oldest *Neothauma* fossil (*Neothauma hattinghi* Van Damme & Pickford, 1999) is reported from the Upper Miocene Kakara Formation from the western branch of the East African Rift System and is 10–11 My old [15,69]. For this second calibration point we used a normally distributed age prior (mean age: 10.5 My, standard deviation: 0.5 My) over the basal node leading to the monophyletic *Neothauma* clade (i.e. stem calibration in BEAST 1.75). Uncorrelated lognormal relaxed clock models were chosen to account for lineage specific rate heterogeneity within each partition; mixing of the Markov chains was monitored with Tracer 1.5 [70].

Ancestral area/state reconstructions as implemented in, e.g., Lagrange, BEAST and Mesquite are not feasible with the present datasets for two reasons. First, the basal topology of the African viviparids is unresolved, whereas a dichotomy is needed to reconstruct the ancestral state and the dichotomy-enforcing algorithm of BEAST would result in low node supports. Secondly, the four major groups of Clade I (Malawi, Congo, Okavango, and Victoria group) form a dichotomous topology (Figure 2), resulting in non-informative ancestral state reconstructions for Split C (Malawi/Congo) and Split B (Okavango/Victoria), and an ambiguous state reconstruction for the most basal Split A due to lacking information (unresolved topology) of the most common recent ancestor.

### Availability of supporting data

The data sets supporting the results of this article are available in the treeBASE repository, http://purl.org/phylo/treebase/phylows/study/TB2:S15381.

## Abbreviations

EARS: East African Rift System; BPP: Bayesian posterior probabilities; cf.: Confer; mrca: Most recent common ancestor; My: Million years; CI: Confidence interval; COI: Cytochrome oxidase subunit I; LSU rRNA: Large subunit of ribosomal RNA.

## Competing interests

The authors declare that they have no competing interests.

## Authors’ contributions

RS analysed the data, developed the model and led the writing of the manuscript. BVB developed the fossil framework of the study and contributed to the model building and writing. FR, TvR, and CA designed and coordinated the study; TvR and CA provided the DNA sequences. Material was collected by FR, BVB, and RS. All authors read and approved the final manuscript.

## Accession numbers

All DNA sequences used in this study are available from NCBI GenBank. Accession numbers are provided in Table 1.

## Supplementary Material

Additional file 1 **The supplementary file contains additional information on the phylogenetic analyses conducted during this study.** We furthermore provide the results of the separate analyses of nuclear and mitochondrial data. Click here for file

Additional file 2 The supplementary file contains additional information on the fossil record of the African Viviparidae and the dating constrains employed in the phylogenetic analysis of this study. Click here for file
