# Supplementary material for: Disjunct distributions of freshwater snails testify to a central role of the Congo system in shaping biogeographical patterns in Africa
Source: BMC Evol Biol. 2014 Mar 6;14:42. doi: 10.1186/1471-2148-14-42 (PMC4015641; doi:10.1186/1471-2148-14-42)

Schultheiß et al.

Disjunct distributions of freshwater snails testify to a central role of the Congo system in shaping biogeographical patterns in Africa

Supplement 1

This supplement contains additional results obtained from phylogenetic analyses of the African Viviparidae.

1. Rates of molecular evolution (i.e. mean substitution rate in percent with confidence interval) and their respective effective sample sizes are provided for each of the four partitions as obtained from the BEAST analyses. Primer details are given in the text. Table S1 additionally contains the number of parsimony informative sites for each partition and the individual partition sizes.

Table S1 – Selected summary statistics from phylogenetic analyses. MSR: mean substitution rate; CI: confidence interval; ESS: effective sample size; PIS: parsimony informative sites.

Partition	MSR	95% CI	ESS	Partition size	PIS
COI	0.93	0.68-1.18	961	593	145
mtLSU rRNA	0.33	0.20-0.47	1100	434	49
ncLSU rRNA	0.059	0.038-0.082	1280	1087	22
Histone 3	0.029	0.013-0.049	4346	328	4

2. We performed the Bayesian analyses additionally for the mitochondrial and the nuclear dataset separately and provide both resulting consensus phylogenies (Figs S1, S2) with their respective support values in the following. Note that the topology in the nuclear phylogeny is very unstable (i.e. the tree shows very low BPP values). This is concordant with the generally low level of parsimony informative sites (see above), which results from the low mean substitution rate. The mitochondrial dataset evolves much faster and the structure of the tree is similar to the phylogeny resulting from the combined analysis of the two datasets.

Figure S1 – Bayesian phylogeny based on the combined nuclear dataset, i.e. ncLSU rRNA and Histone 3. Bayesian posterior probabilities are given for each node. Specimen IDs are identical to the IDs provided in Table 1 in the paper.

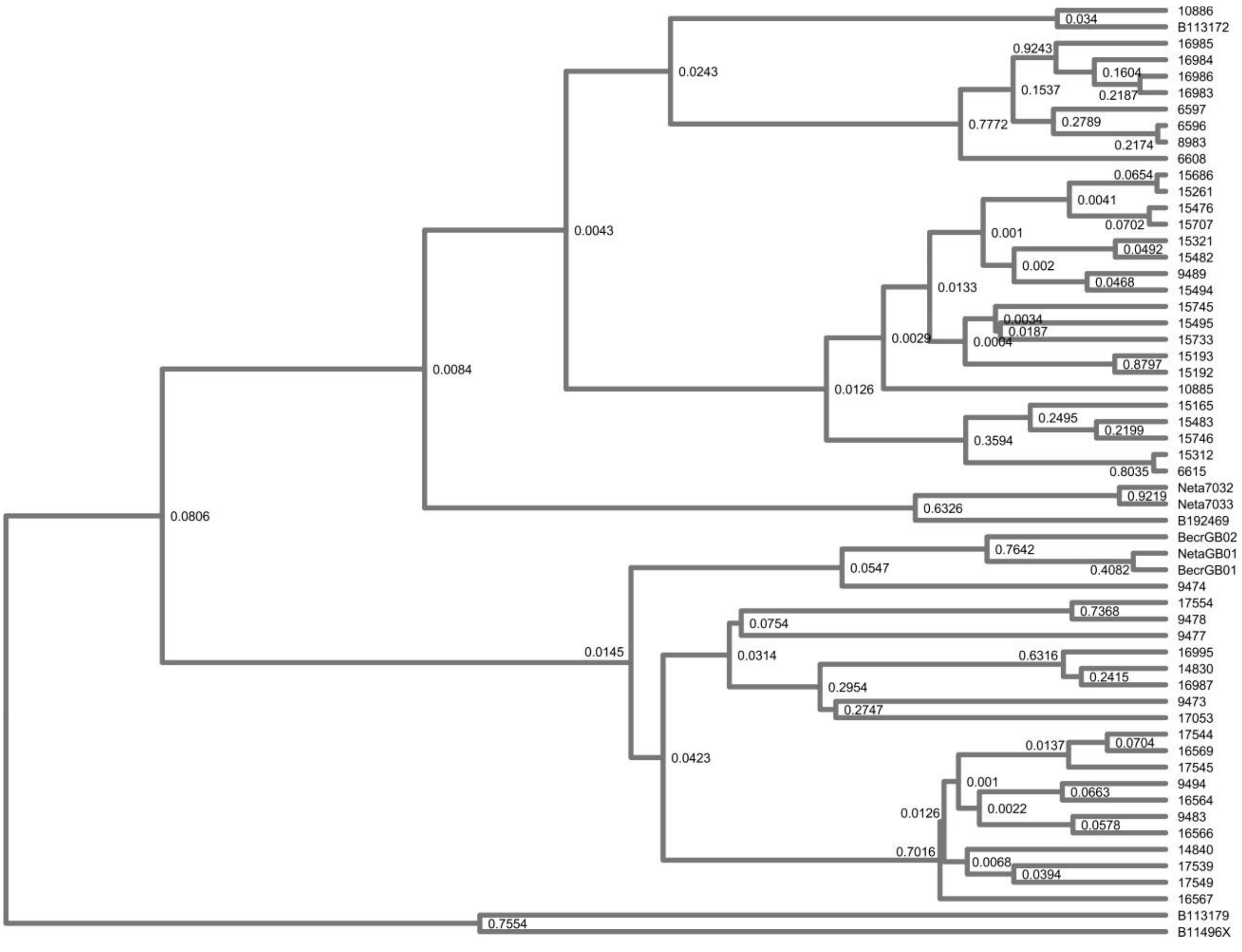

Figure S2 – Bayesian phylogeny based on the combined mitochondrial dataset, i.e. COI and mtLSU rRNA. Bayesian posterior probabilities are given for each node. Specimen IDs are identical to the IDs provided in Table 1 in the paper.

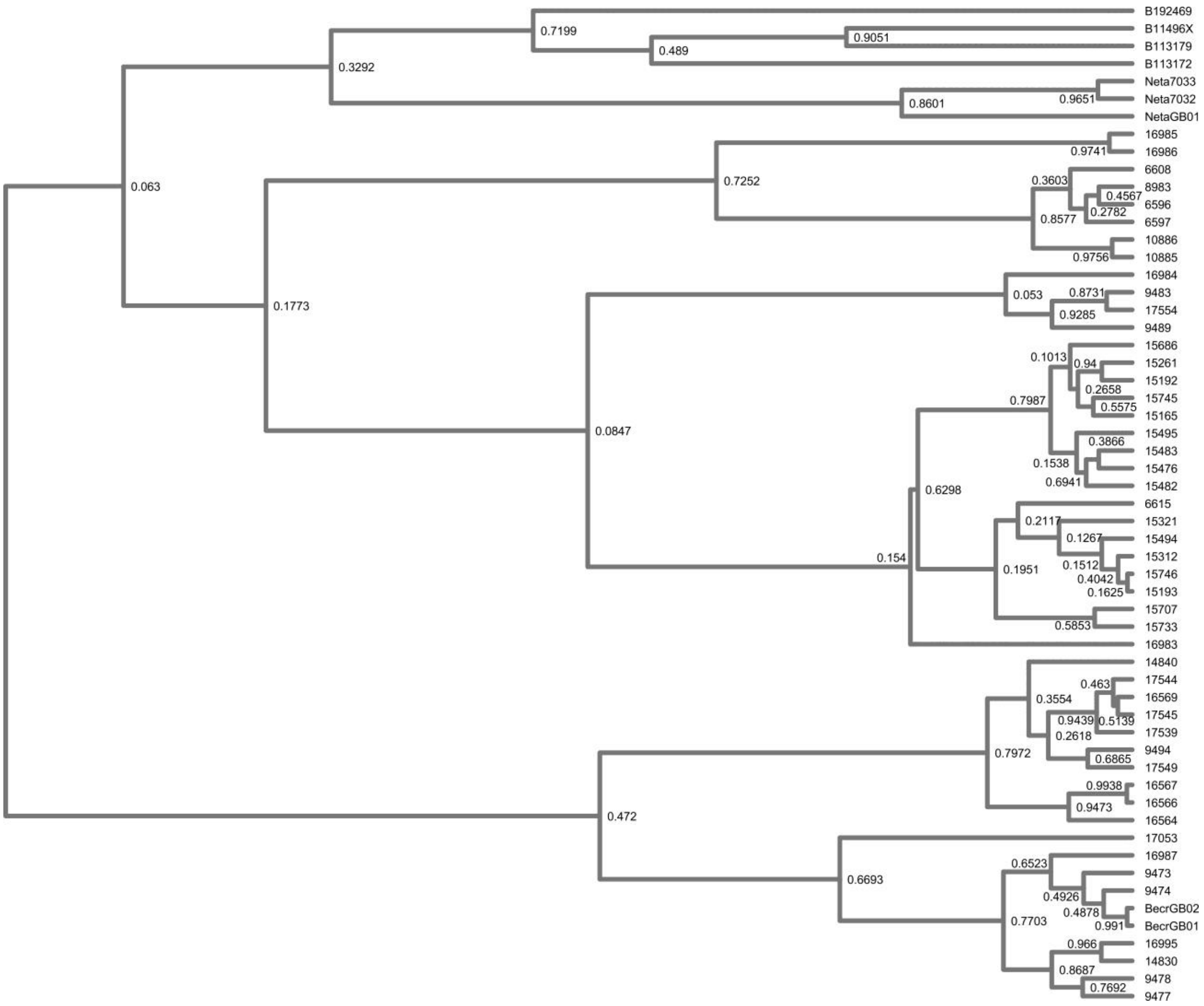

Supplement: Additional file 1 — The supplementary file contains additional information on the phylogenetic analyses conducted during this study. We furthermore provide the results of the separate analyses of nuclear and mitochondrial data. [file 1471-2148-14-42-S1.pdf]
