# Supplementary material for: Disjunct distributions of freshwater snails testify to a central role of the Congo system in shaping biogeographical patterns in Africa
Source: BMC Evol Biol. 2014 Mar 6;14:42. doi: 10.1186/1471-2148-14-42 (PMC4015641; doi:10.1186/1471-2148-14-42)
Supplement: Additional file 2 — The supplementary file contains additional information on the fossil record of the African Viviparidae and the dating constrains employed in the phylogenetic analysis of this study. [file 1471-2148-14-42-S2.pdf]

Schultheiß et al.

Disjunct distributions of freshwater snails testify to a central role of the Congo system in shaping biogeographical patterns in Africa

Supplement 2

The fossil record of African Viviparidae and dating constraints.

All extant African viviparids belong to the subfamily Bellamyinae. Some Cretaceous fossil finds from the Sahara, South Africa and Madagascar were assigned to the family Viviparidae, but the genealogical affinities of these specimens are doubtful [1–4]. These fossils have a *Campeloma*/*Cleopatra*-like shell morphology and if they truly represent viviparids—which has been doubted [5]—they probably belong to another subfamily that became extinct in Africa [4, p. 105]. Hence this material is not informative for the purpose of dating our molecular phylogeny.

The oldest bellamyinid viviparids on the African continent are presumably of Miocene age, but the status and dating of this material has been the subject of considerable debate.

Several authors studied African freshwater molluscs from Early Miocene deposits, e.g. at Rusinga Island, the Turkana Grits and from the Edward-Albert Rift, e.g. [4–7]. Summarizing these reports, Kat [5, p. 739] suggested the presence of diverse Early Miocene mollusc faunas in equatorial East Africa (the current Lake Victoria region) with the notable absence of the bivalve family Unionidae and the gastropod family Viviparidae. More work has been performed since, but Early Miocene viviparids were not found anywhere except for specimens from the Iriri Member at Napak [8,9]. These specimens co-occurred with the bivalve *Iridina moharensis* [8], which was widely distributed over equatorial Africa and is known from deposits with ages

ranging from ~23.0 to 4.7 My [5,10,11]. Deposits at the 'Napak fossil locality I' (Napak Member) are considered to have been formed in the Early Miocene (16-22 Ma) based on early radiometric K-Ar dating [12] and faunal correlations with other sites in East Africa [8,13]. The Iriri Member is more basal in the stratigraphic sequence than the Napak Member, the latter representing more terrestrial environments [8]. We re-examined Pickford's [8] material from the Iriri Member at the Uganda Museum, Kampala, in February 2013 and confirm that these finds are viviparids with a general *Bellamya*-like shape and size (Supplementary Fig. 1). The material is poorly preserved but resembles morphologically the extant *B. unicolor* even though we are reluctant, due to the limited diagnostic features, to support Pickford's [8] claim that it actually belongs to this species.

With respect to the timing of deposition of the Iriri Member at Napak, several aspects are noteworthy. First, the deposits at Napak are considered to occupy essentially the same geographical area and time interval on which Kat and others worked extensively before claiming the absence of viviparids. Second, dating the deposits at Napak has proven to be challenging and absolute age ranges vary widely [12] (Supplementary Table 1). The mammal remains from Napak can be correlated based on faunal affinities to regions with better age control and this in general corroborates an Early Miocene age [13]. However, it does not allow restricting the age range much beyond the ranges provided by absolute dating. Moreover, several authors suggested that bellamyinid viviparids invaded Africa from Asia, e.g. [5], and that this perhaps coincided with the invasion of other faunal elements in the Middle or Late Miocene [14].

The second and third oldest occurrence of bellamyinid fossils are *Bellamya* specimens from deposits at Nyambagasoi and Barsawe in the Baringo Basin, which belong to the Ngorora Formation and are 12.0-11.5 Ma old [10]. Slightly younger are *Neothauma* fossil belonging to *Neothauma hattinghi* from the Kakara Formation of the Albertine Basin (~11.0-10.0 Ma) [9]. Given all the above, it is peculiar that the bellamyinid material at Napak was suggested to be

much older than the beds with subsequent viviparid occurrences. This much older age would imply that the taxon remained geographically restricted for millions of years and that it was not preserved in fossil beds of intermediate ages although such outcrops are abundant in the area around present-day Lake Victoria. To account for the dating uncertainties and in order not to discard any of the previous literature suggestions a priori, we used a wide uniform prior (23.0-13.0 Ma) to calibrate the basal node of our phylogeny using the oldest African bellamyinid.

Supplementary Table 1:

Potassium-Argon determinations from Napak reported by Bishop et al. [12].

Reference	Sample information	Age (Ma)	K2O (%)	Atm. cont. (%)	Remarks
WW 1/2	Biotite from tuff, Napak fossil locality I	19.0±2.0	6.2	66.5	Obtained by Damon; see Bishop [15]
WW 1/11	Biotite from coarse tuff, Napak fossil locality I	25.0±1.8	3.66	n.a.	Considered discrepantly high due to the presence of mica
MB/1	Coarse (?welded) tuff underlying Napak fossil locality I by ~150m; total rock analysis, Napak II	17.8±0.5	1.61	78.1	Sample may have been emplaced by ash-flow; age is considered to be the minimum age for emplacement of tuff; stratigraphically older than MB/23, SUN 1, SUN 2, MB/3 and WW1/2
MB/3	Mica from tuff, Napak I	17.8±0.4<br>17.8±0.4<br>17.8±0.4	6.03<br>6.03<br>6.03	82.1<br>82<br>81	Ages considered discrepantly low; Damon's ages for WW 1/2 are preferred over these dates by Bishop et al. [12]
MB/4	Mica from tuff, Napak IX	14.5±0.6	7.14	78.6	
MB/23	Melanelinite lava, Napak I	12.8±0.5<br>7.5±0.5	1.56<br>1.56	82<br>95	these are minimum ages that were considered discrepant by Bishop et al. [12]
SUN 1	Nephelinite lava, Irire, Napak, Ground sample	14.3±0.7<br>6.7±1.5<br>6.7±1.5	1.68<br>1.68<br>1.84	83.1<br>86<br>90.6	Considered stratigraphically equivalent to MB/23 based on Trendall [16] by Bishop et al. [12]; ages widely discrepant, dates anomalous
SUN 2	Nephelinite lava, Irire, Napak, Ground	27.5±2.6	1.71	71.9	Considered stratigraphically equivalent to MB/23 based

	sample	18.7±2.0	1.71	68.7	on Trendall [16] by Bishop et al. [12]; ages widely
		14.2±1.3	1.83	72.7	discrepant, dates anomalous
SUN 159	Nepheline from ijolite, Lokupoi, Napak	23.2±1.4	6.35	68.4	
		24.0±1.3	6.35	65.5	
		24.5±1.2	6.35	63.3	
SUN 30	Nepheline from ijolite, Lokupoi, Napak	29.2±1.3	5.92	69.6	Minor alteration, probably true age of crystallization
		30.8±1.4	5.92	68	
SUN 155	Nepheline from ijolite, Lokupoi, Napak	30.8±1.0	6.15	65.1	Minor alteration, probably true age of crystallization
		31.3±1.0	6.15	63.7	

Footnote: Sample MB/1 comes from the base of the Iriri Member; MB/23, SUN 1 & SUN 2 from tuffs younger than the Iriri Member, but older than the Napak Member; MB/3, WW1/2, WW1/11 from the Napak Member; MB/4 from the top of the Napak Member; SUN 159, SUN 30, SUN 155 come from the compact igneous plug from the main Napak volcano vent; WW1/2 is considered the most reliable date for the Napak Member by Bishop et al. [12]. Abbreviation: Atm. cont.—atmospheric contamination.

Supplementary Figure 1

Fossil *Bellamya* specimen from the Iriri Member of the Napak Formation at Napak. This material was described but not depicted by Pickford [8]. Scale bar is 10 mm.

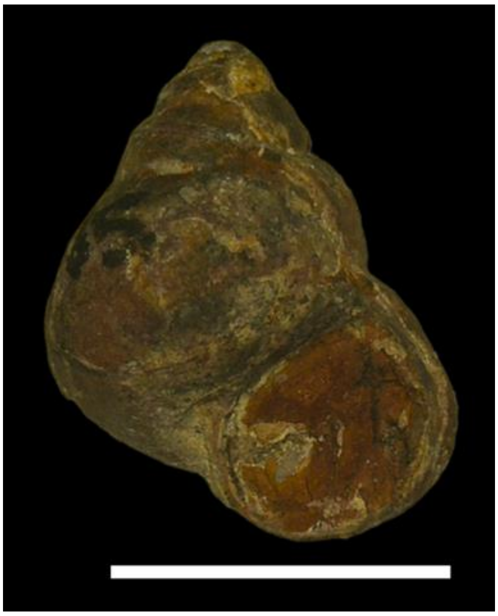

References.

1 Fischer JC: **Les mollusques du 'Continental intercalaire' (Mésozoïque) du Sahara Central, Gastéropodes**. *Mémoires de la Société géologique de France 1963*, **96**:41–52.

2 Newton RB: **On some freshwater fossils from central South Africa.** *The Annales and Magazine of Natural History 5 1920*, **9**:241–249.

3 Prashad B: **Recent and fossil Viviparidae. A study in distribution, evolution, and palaeogeography.** *Memoirs of the Indian Museum 1928*, **8**:153–251.

4 Van Damme D: *The freshwater Mollusca of Northern Africa: distribution, biogeography, and palaeoecology*. W. Junk; 1984.

5 Kat PW: **Biogeography and evolution of African fresh-water mollusks – implications of a Miocene assemblage from Rusinga-Island, Kenya.** *Palaeontology 1987*, **30**:733–742.

6 Gautier A, Van Damme D: **A revision of the Miocene freshwater mollusks of the Mohari Formation (Sinda-Mohari, Ituri, N. E. Zaïre).** *Annals of the Royal Museum for Central Africa, serie 8, 1973*. **45**:43–62.

7 Verdcourt B: **The Miocene non-marine Mollusca of Rusinga Island, Lake Victoria and other localities in Kenya.** *Palaeontographica 1963*, **121**:1–37.

8 Pickford M: **Palaeoenvironments of Early Miocene hominoid-bearing deposits at Napak, Uganda, based on terrestrial molluscs**. *Annales de Paléontologie 2004*, **90**:1–12.

9 Van Damme D, Pickford M: **The late Cenozoic Viviparidae (Mollusca, Gastropoda) of the Albertine Rift Valley (Uganda-Congo).** *Hydrobiologia 1999*, **390**:171–217.

10 De Groeve E: *The late Cenozoic freshwater molluscs of the Tugen Hills (Kenya): taxonomy, paleoecology and paleozoogeography*, 2005.

11 Van Damme D, Pickford M: **The Late Cenozoic bivalves of the Albertine Basin (Uganda-Congo).** *Geo-Pal Uganda 2010*, **2**:1–121.

12 Bishop WW, Miller JA, Fitch FJ: **New Potassium-Argon Age determinations relevant to the Miocene fossil mammal sequence in East Africa.** *American Journal of Science 1969*, **267**:669–699.

13 Pickford M: **Preliminary Miocene mammalian biostratigraphy for Western Kenya.** *J. Hum. Evol. 1981*, **10**:73–97.

14 Van Damme D, Van Bocxlaer B: **Freshwater molluscs of the Nile basin, past and present**. In *The Nile – Origin, Environments, Limnology and Human Use*. Edited by Dumont HJ. Springer Netherlands; 2009.

15 Bishop, WW: **More fossil primates and other Miocene mammals from northeast Uganda.** *Nature 1964*, **203**:1327–1331.

16 Trendall AF: *Explanation of the geology of sheet No. 35* (Napak), .
